# The Injury Mechanism of Traumatic Amputation

**DOI:** 10.3389/fbioe.2021.665248

**Published:** 2021-04-15

**Authors:** Iain A. Rankin, Thuy-Tien Nguyen, Louise McMenemy, Jonathan C. Clasper, Spyros D. Masouros

**Affiliations:** ^1^Department of Bioengineering, Imperial College London, London, United Kingdom; ^2^Academic Department of Military Surgery and Trauma, Royal Centre for Defence Medicine, ICT Centre, Birmingham Research Park, Birmingham, United Kingdom; ^3^Department of Trauma and Orthopaedic Surgery, Frimley Park Hospital, Surrey, United Kingdom

**Keywords:** biomechanics, traumatic amputation, fracture, blast injury, military, mouse, soil, sand

## Abstract

Traumatic amputation has been one of the most defining injuries associated with explosive devices. An understanding of the mechanism of injury is essential in order to reduce its incidence and devastating consequences to the individual and their support network. In this study, traumatic amputation is reproduced using high-velocity environmental debris in an animal cadaveric model. The study findings are combined with previous work to describe fully the mechanism of injury as follows. The shock wave impacts with the casualty, followed by energised projectiles (environmental debris or fragmentation) carried by the blast. These cause skin and soft tissue injury, followed by skeletal trauma which compounds to produce segmental and multifragmental fractures. A critical injury point is reached, whereby the underlying integrity of both skeletal and soft tissues of the limb has been compromised. The blast wind that follows these energised projectiles completes the amputation at the level of the disruption, and traumatic amputation occurs. These findings produce a shift in the understanding of traumatic amputation due to blast from a mechanism predominately thought mediated by primary and tertiary blast, to now include secondary blast mechanisms, and inform change for mitigative strategies.

## Introduction

Recent conflicts have seen improvised explosive devices (IEDs) rise as the insurgents’ weapon of choice, where they have been the primary cause of military deaths (Clasper and Ramasamy, 2013). Outside of the military setting, use of IEDs by terrorist organisations has increased steadily over the last 40 years (Edwards et al., 2016). One of the most common and defining injuries of an IED explosion is of blast-mediated traumatic amputation (Ramasamy et al., 2009a). This injury represents a significant cause of morbidity and mortality. It is associated with fatality either directly through haemorrhage, or indirectly as a marker of other severe blast trauma (Mellor and Cooper, 1989). With regards to morbidity, a US-Army study showed only a 2.3% return-to-duty rate for soldiers who had sustained a traumatic amputation (of whom most had suffered only partial hand or foot loss) (Kishbaugh et al., 1995). The 2013 Boston Marathon bombing caused 17 lower limb traumatic amputations and a further 10 severe soft tissue extremity injuries (King et al., 2015); the morbidity in these civilian injuries is likewise extensive with reduced mobility, phantom limb pain, and an overall reduced quality of life reported (Sinha et al., 2011; Azocar et al., 2020). To limit future morbidity and mortality through mitigative strategies, an accurate understanding of the mechanism of injury is essential.

The mechanisms of injury due to an explosion in general can be separated into five distinct categories: primary (direct effects of the shock front over-pressurisation), secondary (injury caused by energised projectiles propagated by the blast), tertiary (bodily displacement, either directly or indirectly as a result of the blast wind), quaternary (a miscellaneous category of injuries, including burns), and quinary (non-explosion related effects resulting in a hyper-inflammatory state, including through the use of biological, chemical or nuclear products). The mechanism of injury by which blast results in traumatic amputation is not clearly understood. Several mechanisms of injury have been proposed. The first proposed mechanism was hypothesised to occur due to a combination of primary and tertiary blast mechanisms, whereby the shock front over-pressurisation causes diaphyseal fracture through shear and axial stress, followed by the blast wind completing the amputation (Hull and Cooper, 1996). Other proposed mechanisms of injury include tertiary blast injury in isolation, as rapid lower limb movement propagated by the blast wind results in traumatic amputation (Singleton et al., 2014). Secondary blast injury has also been linked to traumatic amputation, where single large fragments propagated by the blast have resulted in “guillotine type” injuries (Hull and Cooper, 1996). More recently, we have shown secondary blast injury as a result of energised environmental debris to be linked to causing traumatic amputation in an animal model (Rankin et al., 2020a). Whilst we showed that high velocity environmental debris (sandy gravel soil) can cause a cohort of injuries, including traumatic amputation, the exact mechanism by which the traumatic amputation had occurred was not examined specifically.

With regards to the type of environmental debris, North Atlantic Treaty Organization (NATO) standards for testing protection against a buried explosive device defines the testing conditions as utilising a soil type which is of a sandy gravel composition (Nato/PfP Unclassified, 2006). Whilst the mechanism by which energised sandy gravel soil causes traumatic amputation is not clear, the process by which it propagates following an explosion is known. When a buried explosive is detonated, the resultant shockwave compresses this surrounding sandy gravel soil. Immediately following this, gas from the explosion is released at high velocity and acts to eject this compressed soil at supersonic speeds, which rapidly decelerate to below 600 m/s before impacting with casualties (Bowyer, 1996; Tremblay et al., 1998). The soil is carried upwards from the ground by the gas flow to project, dependent upon the soil’s characteristics, at an angle of between 45 and 120 degrees, in a cone shape. With dry soil, easier venting of gaseous detonation products results in a wider spread. In contrast, water saturated soil resists gaseous venting to a greater degree; this results in a tunnelling effect and concentration of the soil in a vertical direction, which may result in increased injury at the point of impact (Grujicic et al., 2008; Ramasamy et al., 2009b). This injury mechanism has previously been referred to as “sand blast” (Webster et al., 2018).

A further variable which may affect injury risk is the size of the propagated soil. Typical sandy gravel soil granulometry has been described, with ideally distributed particle sizes ranging from 0.1 to 40 mm (Nato/PfP Unclassified, 2006). The effect that variations in soil size or moisture content may have on the injury risk of traumatic amputation is not known.

The aims of this study were (1) to replicate isolated traumatic amputation in a cadaveric small animal mouse model, caused by propagated high velocity sandy gravel soil (subsequently referred to as “sand blast”), (2) to investigate and describe the mechanism of injury of sand blast mediated traumatic amputation, through high-speed video recording and injury documentation, and (3) to investigate the effect of changes in sandy gravel soil size and moisture content on the risk for sustaining traumatic amputation.

## Materials and Methods

The experimental design and procedures were carried out in compliance with the UK Animal (Scientific Procedures) Act 1986. Testing was conducted using an established model on fresh-frozen cadaveric male MF-1 (out-bred, ex-breeder, wild type) murine specimens (8–9 weeks of age, Charles River Ltd., United Kingdom) (Rankin et al., 2019). Specimens were stored at −20°C and thawed at room temperature (21 ± 2°C) prior to testing.

Sandy gravel soil sizes were chosen based upon NATO unclassified AEP-55 recommendations for typical sandy gravel soil granulometry (Nato/PfP Unclassified, 2006). This was subsequently scaled to the murine model based upon recommended animal scaling parameters in blast, where the scale is equal to the length of a parameter of the human species divided by that of the animal species used (λ_*L*_ = L_1_/L_2_) (Panzer et al., 2014). The thigh circumference of each species was taken as the representative parameter for scaling, in view of traumatic amputation of the lower limb as the primary outcome. Median mouse thigh circumference was calculated as 2.7 cm (range 2.4–3.2 cm) from specimens (*n* = 59), whilst human thigh circumference was taken from literature as 55 cm (White and Churchill, 1971). From this, a downscaling of 20× for sandy gravel size was utilized (λ_*L*_ = 55 / 2.7 = 20). A minimum sandy gravel size cut-off of 0.1 mm was taken to avoid sublimation of sandy gravel particles smaller than this at high velocity.

Testing with different sandy gravel soil size and moisture content was performed to ascertain for any difference seen in injury risk. Three sandy gravel soil size ranges were tested, consisting of (1) ideally distributed, (2) minimum, and (3) maximum sandy gravel soil size range. These groups were further subdivided into dry, or saturated with water prior to testing. Sand saturated with water was formed by first submerging a sample of dry sand into a beaker of shallow water (with sufficient quantity to cover the total sand mass). The sand was then removed from the beaker by means of a laboratory micro spatula and transferred to absorbent tissue paper, to remove excess water. The sand was subsequently transferred from the tissue paper via micro spatula to the hollow polycarbonate sabot, for use immediately in an experiment.

**FIGURE 1:**
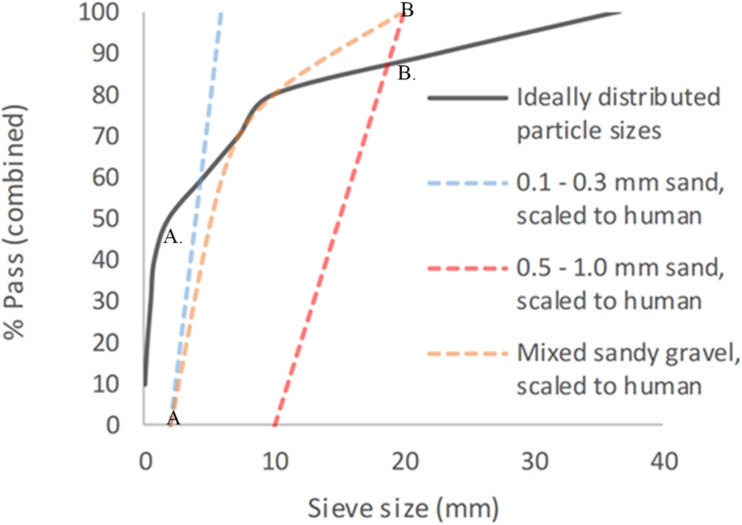
Experimental sandy gravel sizes used, scaled to human values, shown alongside ideally distributed particle sizes. (A) = human median value. (B) = human 85th centile. (A) = lower limit of experimental sandy gravel range. (B) = upper limit of experimental sandy gravel range. % pass (combined) describes the percentage of total volume of sandy gravel passing a specific sieve size; sieve size (mm) relates to the diameter of each hole within the sieve.

This gave a total of six different sandy gravel soil test groups. The ideally distributed sandy gravel soil size range chosen consisted of sandy gravel as closely representative to human scaled values, ranging from the human ideal particle size median value to the 85th centile value, consisting of 60% sandy gravel sized 0.1 to 0.3 mm, 20% sized 0.3 to 0.5 mm, and 20% sized 0.5 to 1 mm. The minimum sandy gravel soil size group consisted of 100% sandy gravel sized 0.1 to 0.3 mm. The maximum sandy gravel soil size group consisted of 100% sandy gravel sized 0.5 to 1 mm. The experimental sand sizes and distribution used (scaled to human values) are shown alongside those recommended in NATO AEP-55, ideally distributed particle sizes in Figure 1 (Nato/PfP Unclassified, 2006).

The sandy gravel was housed within a hollow polycarbonate sabot which was loaded into the firing chamber of a double-reservoir gas-gun system (Nguyen et al., 2018). Within this system, a 2-litre reservoir charged with air or helium and a Mylar^®^ diaphragm firing mechanism was used to accelerate the sabot-sand unit down a 3-m-long, 32-mm-bore barrel. The output velocity was controlled by the thickness of the Mylar^®^ diaphragm. The reservoir section of the gas gun was charged to a predetermined firing pressure, to accelerate the sabot-sand unit to the desired velocity. The pressure was maintained within the reservoir section by a Mylar^®^ diaphragm of appropriate thickness (ranging from 50 to 150 μm). The system utilises a priming section, which is charged to a pressure below the rupture pressure of the diaphragm. This reduces the pressure gradient across the mylar diaphragm (containing the reservoir system) and prevents it from rupturing early, as the reservoir is filled. The pressure in the prime section is vented at the point of initiating firing of the gas gun, resulting in rupture of the diaphragm, with release of the pressurised gas. The gas-gun system accelerates the sabot-sandy-gravel unit down a barrel to exit into a target chamber, where the sabot is separated from the sandy gravel by a sabot stripper. The sabot is halted at this point, while the sandy gravel continues to travel toward the mouse specimen at the intended terminal velocity.

**FIGURE 2:**
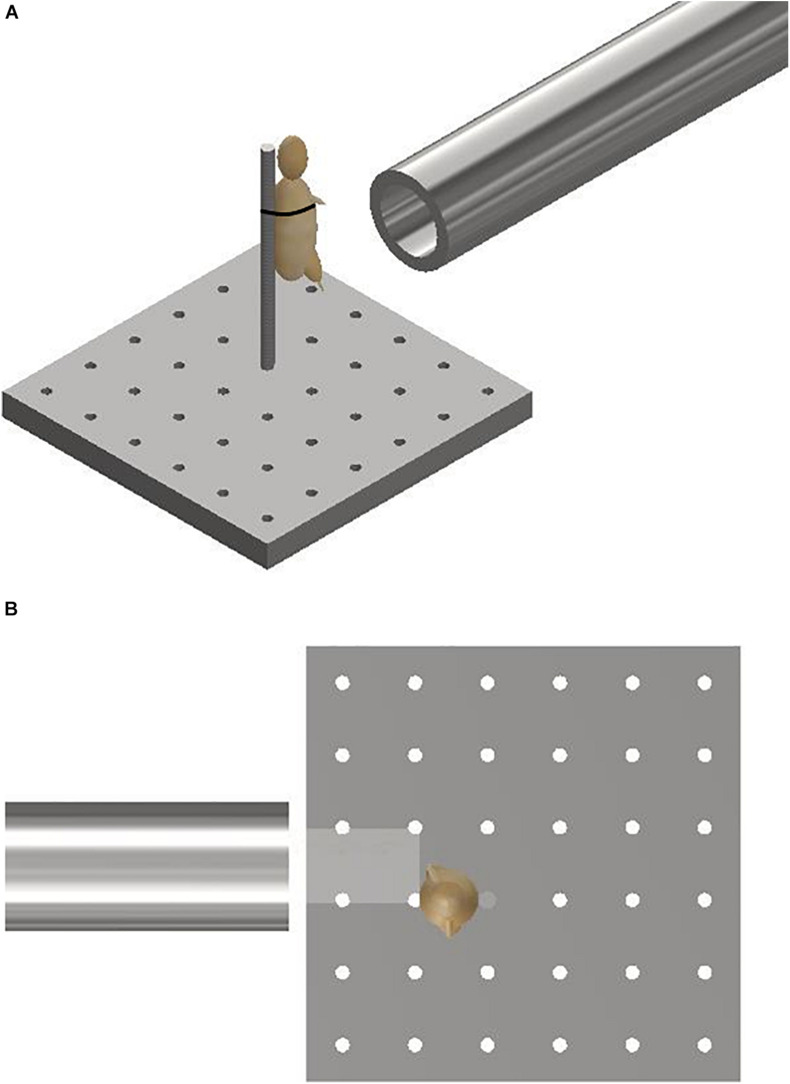
**(A)** Schematic illustrating the experimental setup showing the gas-gun outlet with mounting platform and mouse. The mouse represented with a model. **(B)** Aerial view of schematic illustrating initial sandy gravel stream passing through distal outlet to impact with offset lower limb of mouse. The mouse is represented with a model.

Mice were secured in an upright posture on a steel mount of 10 mm diameter fixed within the target chamber, 50 mm distal to the gas-gun outlet. A single cable tie across the thorax was applied to secure the specimens in position on the mount, whilst leaving the lower limbs exposed and freely mobile (Figure 2). The right lower limb was centred in the midpoint of the path of the focused sand blast. Experiments were then repeated with re-positioning of the mount to target the contralateral limb.

The speed of the sandy gravel particles at the point of impact with the specimen was estimated using high-speed photography (Phantom VEO710L, AMETEK, United States) at 68,000-fps. An average velocity for the sand blast was determined based upon identifying and tracking four unique points evenly distributed across the sandy gravel. From this, the mean with standard deviation of the velocity of the sand blast as a whole was calculated.

A single control test was performed utilising the maximum gas-gun pressure used previously with the absence of any sandy gravel ejecta. This was performed in order to ascertain whether any injurious effects are caused by the pressurised air alone. This control test was performed on a single mouse specimen.

Prior to and following each test, mouse specimens underwent radiographic imaging using a mini C-arm (Fluoroscan^®^ InSight^TM^ FD system, United States) to identify any lower limb fractures. Following this, the specimens were reviewed to identify lower limb traumatic amputation. Where a lower limb open fracture was present with extensive soft tissue loss, the injury was classified as a traumatic amputation.

### Statistical Analysis and Development of the Risk Function

The NCSS statistical software was used for statistical analysis (version 12, UT, United States). A likelihood-criteria best-fit analysis, with the aid of probability plots, was performed to choose the distribution that best fit the data for each injury type. The Weibull distribution was shown to be the best fit in the majority of cases; hence, it was chosen as the probability distribution to represent the risk for all injury types observed in this study. Weibull survival analysis was used to examine the association between sandy gravel velocity and traumatic amputation. The Weibull regression model is *P*(*v*) = 1 − *e*^−(*v*/λ)^κ^^, where *P* is the probability of injury, *v* (the average velocity of the sandy gravel) is the predictor variable, and *λ* and *κ* are the corresponding coefficients associated with the predictor variable. To derive the injury-risk curves, data were classified as left censored where injury was present and right censored where there was no injury. A *post hoc* two-sample Kolmogorov-Smirnov test was performed to assess for significant differences between the distribution of injury-risk curves across groups. A Bonferroni corrected α value of 0.0083 was used to compensate for multiple comparisons (0.0083 = 0.05/6).

## Results

**FIGURE 3:**
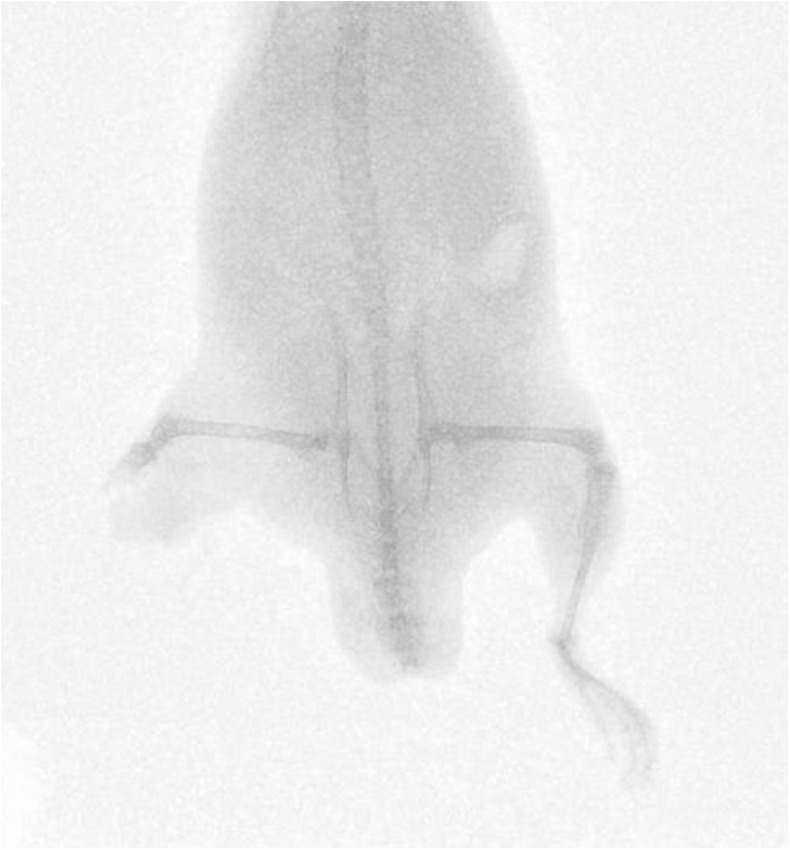
Radiograph of mouse injured with high velocity sand blast, sustaining a right sided lower limb traumatic amputation.

**FIGURE 4:**
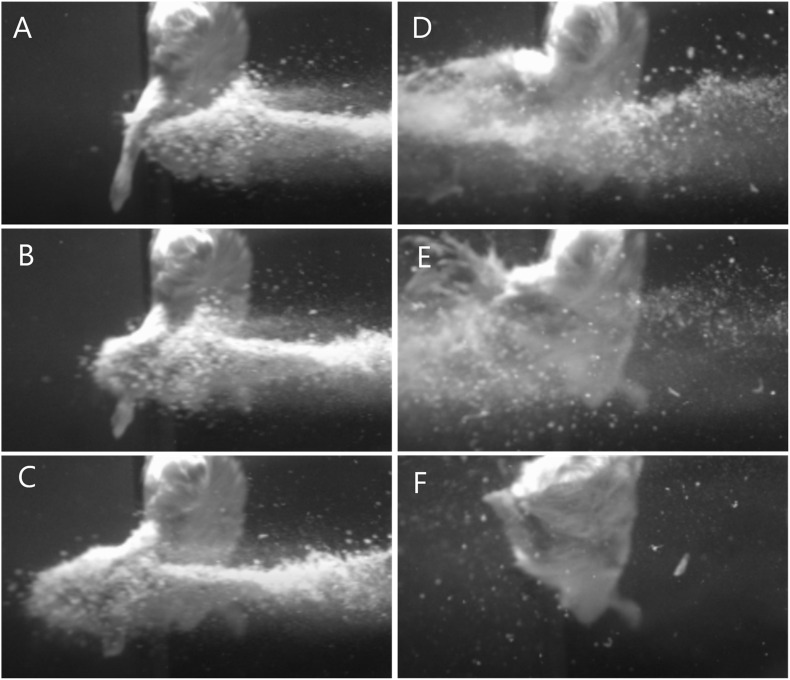
Images illustrating the stages of traumatic amputation secondary to high velocity sand blast. **(A)** Immediately pre-impact. **(B)** Point of initial impact. The sandy gravel has begun to move through and around the tissues of the lower limb at high velocity. Due to the experimental setup the foot has evaded the trajectory of the sandy gravel, whilst the limb above has begun to fragment and displace relative to the foot below. **(C)** The foot has been pulled upward into the trajectory of the sandy gravel, whilst the skeletal and soft tissue above are now significantly fragmented and displaced. **(D)** The lower limb has now been entirely shattered and displaced, with soft tissue stripping on the periphery of the blast now evident as the muscle is seen moving outwards. **(E)** As the sand blast dissipates, the remaining surrounding soft tissues can be seen more clearly to be stripped and displaced. **(F)** Completed traumatic amputation. (A schematic to provide context of the animal’s position and orientation is provided in Figures 2A,B).

Fifty-nine cadaveric mice were used across experiments, comprising of a total of 117 lower limbs impacted by high-velocity sandy gravel soil, and one lower limb control specimen. No injuries were seen in the control specimen. A gas-gun system was used to accelerate the sandy gravel; the average sand blast velocity at the exit of the gun’s barrel ranged from 20 ± 5 to 136 ± 5 m/s. A radiograph showing a mouse which sustained a traumatic amputation due to high velocity sand blast is shown in Figure 3. Supplementary Material Video 1 shows a 68,000 frames per second (fps) recorded video with an aerial viewpoint, played at 30 fps, capturing sandy gravel soil travelling at 64 m/s as it impacts a specimen. Supplementary Material Video 2 shows a 68,000-fps recorded video with a side-on viewpoint, played at 30 fps, capturing sandy gravel impact at 130 m/s. Images from Supplementary Material Video 2, showing the sequential stages of sand blast impact, can be seen in Figure 4.

**FIGURE 5:**
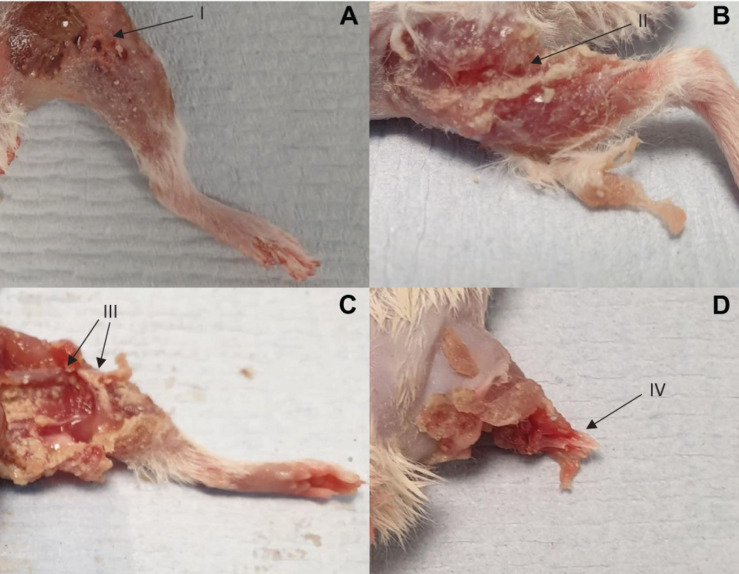
Four separate injuries of worsening severity sustained following impact with high velocity sand blast. **(A)** Burst lacerations and skin tears seen at I. **(B)** Involvement of the underlying subcutaneous and muscular layers, with muscle tears and stripping seen at II. **(C)** Associated open segmental femoral fracture seen at III, with extensive surrounding soft tissue damage and loss. **(D)** Complete limb avulsion with traumatic amputation seen at IV.

Images showing exemplar injuries sustained are shown in Figure 5. These images show increasing severity of injury: initial skin lacerations and superficial wounding only (A), skin and underlying soft tissue injury (B), associated open fracture with extensive tissue loss (C), and complete limb avulsion (D).

**FIGURE 6:**
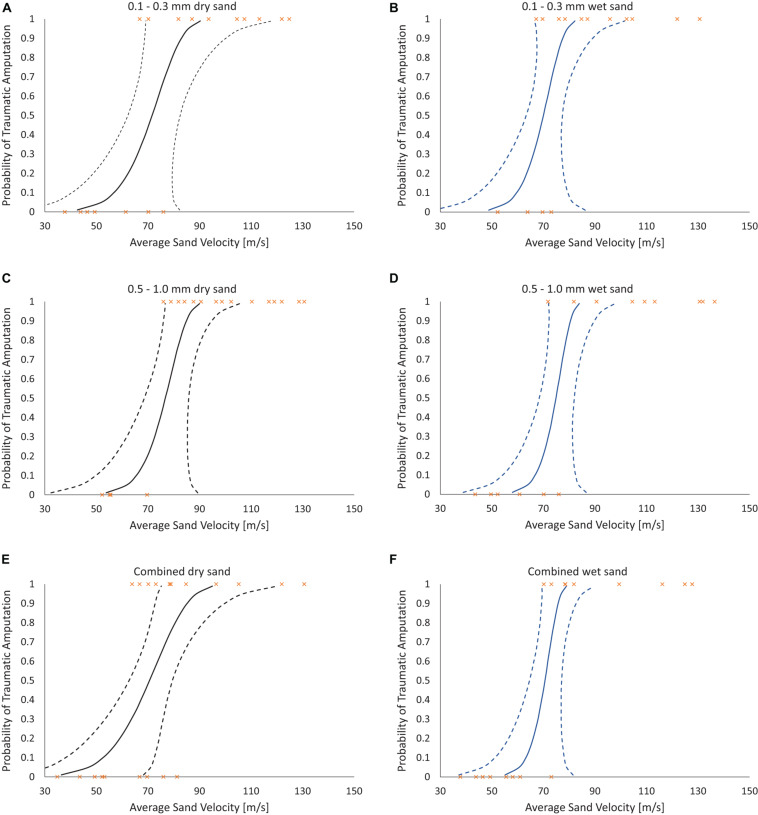
Traumatic amputation (as per Figures 5C,D) risk curves as a function of average sandy gravel velocity. **(A)** 0.1–0.3 mm dry sandy gravel. **(B)** 0.1–0.3 mm wet sandy gravel. **(C)** 0.5–1.0 mm dry sandy gravel. **(D)** 0.5–0.1 mm wet sandy gravel. **(E)** Combined (ideally distributed) dry sandy gravel. **(F)** Combined (ideally distributed) wet sandy gravel. 95% CI represented with dashed lines.

Risk of traumatic amputation increased with increasing sand blast velocity across all groups. The 50% risk of traumatic amputation ranged from 70 m/s (95% CI 63–77 m/s) in the 0.1–0.3 mm wet sandy gravel group to 77 m/s (95% CI 69–86 m/s) in the 0.5–1.0 mm dry sandy gravel group. No significant differences between the distribution of injury-risk curves for sandy gravel soil groups were seen, including across size ranges and moisture content (Table 1). Full injury risk curves with 95% CIs are shown in Figure 6, with the 25, 50, and 75% risks of injury presented in Table 2.

**TABLE 1:** Two sample Kolmogorov-Smirnov test to assess for significant differences between the distribution of injury risk curves.

	0.1–0.3 dry	0.5–1.0 dry	Mix dry	0.1–0.3 wet	0.5–1.0 wet
0.1–0.3 dry					
0.5–1.0 dry	0.591				
Mix dry	1.000	0.358			
0.1–0.3 wet	0.841	0.095	0.841		
0.5–1.0 wet	0.591	0.841	0.358	0.194	
Mix wet	0.591	0.841	0.358	1.000	0.194

**P* values shown.*

**TABLE 2:** The velocities (m/s) at 25, 50, and 75% risk of injury (V_25_, V_50_, and V_7__5_, respectively) for traumatic amputation across all group.

	V_25_ (95% CI) m/s	V_50_ (95% CI) m/s	V_75_ (95% CI) m/s
0.1–0.3 mm, dry	64 (52–80)	72 (63–83)	78 (67–90)
0.5–1.0 mm, dry	71 (60–85)	77 (69–86)	81 (74–89)
Ideally distributed, dry	62 (50–75)	71 (63–80)	79 (70–89)
0.1–0.3 mm, wet	65 (55–77)	70 (63–77)	74 (67–82)
0.5–1.0, wet	71 (62–81)	75 (68–82)	78 (72–85)
Ideally distributed, wet	67 (58–77)	71 (65–77)	74 (68–79)

*95% confidence intervals (CI) in parenthesis.*

## Discussion

The first aim of this study was to reproduce isolated traumatic amputation due to sand blast in a cadaveric mouse model, utilising a gas-gun system. We showed that high velocity sand blast is an independent mechanism of injury causing traumatic amputation, with extensive soft tissue and skeletal disruption seen at high velocities. The injury curves presented (Figure 4) show a clear link between increasing sandy gravel velocity and likelihood of injury. For example, ideally distributed dry sandy gravel showed a 25, 50, and 75% risk of traumatic amputation at sand blast velocities of 62, 71, and 79 m/s, respectively.

We have previously demonstrated traumatic amputation in conjunction with pelvic fractures, perineal injury and open abdominal trauma, due to impact with a widely dispersed cloud of high velocity sandy gravel, from an under-body blast position (Rankin et al., 2020a). High velocity sand blast was implicated in the mechanism of injury for traumatic amputation, however, from the injury outcome data alone a characterisation of the process was not possible. In the present study, we have utilised a focused sand blast to impact the lower limb in isolation. This has allowed us to characterise the pattern and development of injury and provide a detailed account of the underlying mechanism of injury. Based on these findings, we describe in detail and characterise the process of traumatic amputation due to high velocity sand blast: an initial bolus of compressed sandy gravel soil is propagated at high velocity toward the casualty (Figure 4A). The initial impact results in superficial burst lacerations and tears to the skin of impacted limbs (Figure 5A). As the soil continues to propagate (Figure 4B), it progresses to infiltrate deep to the skin, spreading out both within and through tissue planes; this occurs through a series of multiple microtraumas to the underlying fascia and muscular tissue, where the sand blast damages and displaces these soft tissues (Figure 5B). With sufficient velocity, the soil progresses to cause a series of microfractures to the underlying skeletal structures which compound to cause segmented or multifragmentary fractures to the long bones of the lower limb; the ongoing impact of soil to the soft tissues of the limb has at this stage resulted in extensive soft tissue loss in association with long bone fractures (Figure 5C). The skeletal and soft tissue are now seen to be fragmented and displaced (Figure 4C). A critical injury point is reached, whereby the underlying integrity of both skeletal and soft tissues of the limb has been compromised (Figure 4D). These tissues progress to be avulsed, whilst tissues in the periphery are injured and propagated outward from the point of maximal impact (Figure 4E). At this stage, a completed traumatic amputation of the limb has occurred (Figures 4F, 5D).

Multiple mechanisms of injury for blast-related traumatic amputation have been described. The initial accepted mechanism of injury was hypothesised to be due to the initial blast shock front causing a diaphyseal fracture of the limb, with the subsequent blast wind separating and amputating the limb at the point of fracture. This theory was based on laboratory work with a goat hind limb model, which showed that a diaphyseal fracture occurred when a long bone was impacted with a shock front but shielded from the subsequent blast wind or any associated secondary blast injury (Hull and Cooper, 1996). Of note, diaphyseal fracture occurred at distances of 0.5 m proximity to the explosive, but not at 1 m, suggesting the requirement for the casualty to be in close proximity to the explosive for this mechanism of injury to occur (Hull and Cooper, 1996). Further underpinning this mechanism was the clinical association at the time of traumatic amputation to fatal traumatic blast-lung injury, and a lack of through-joint traumatic amputations (Mellor and Cooper, 1989; Hull et al., 1994). More recent military data have questioned this theory. Data from the recent conflicts in Iraq and Afghanistan showed no link between traumatic amputation and primary blast-lung injury, with a high proportion of amputees surviving their injuries; furthermore, a substantially higher incidence of through-joint traumatic amputation was seen, again questioning the shockwave mediated diaphyseal fracture mechanism of injury (Singleton et al., 2014). In that study, we hypothesised that the blast wind played a far more substantial role in the mechanism of injury for traumatic amputation and could itself be a mechanism of injury independent of other factors (Singleton et al., 2014).

Our previous work investigating pelvic fracture and vascular injury due to a shock-tube mediated blast wave (consisting of both a shock front and subsequent blast wind) using a cadaveric mouse model, showed traumatic amputation rates following blast far lower than what would be expected to be present in association with the pelvic fractures and vascular injury seen, as compared to battlefield data (Rankin et al., 2019, 2020b). We subsequently showed that when an initial injuring force to the lower limb occurred prior to impact with the blast wind, traumatic amputation occurred. We concluded that the lower-than-expected traumatic amputation rates were likely due to the absence of any secondary blast injury from the experimental model, to cause this initial injury (Rankin et al., 2019). The current study has shown that high velocity sand blast (a secondary blast-injury mechanism) can be in and of itself, an independent mechanism of injury causing traumatic amputation. Both shock tube and gas-gun experimental models are surrogates of the blast environment. Both platforms provide parts of the blast injury in isolation: a shock-tube system allows focused study of the shock front and blast wind (primary and tertiary blast injury) whilst the gas-gun system allows focused study of energised environmental debris (secondary blast injury). Both platforms have produced traumatic amputation, of varying incidence rates, in a cadaveric animal model. In a blast environment, all of these mechanisms (the primary shock front, the secondary energised environmental debris, and the tertiary blast wind causing bodily displacement) occur together. As such, whilst each is possible of causing traumatic amputation in isolation, the reality likely is that traumatic amputation is caused by all three of these described mechanisms synergistically, to varying degrees of each, dependent upon the blast conditions. These mechanisms acting synergistically are thought to be the causative factors for both military and civilian blast-mediated traumatic amputation, where in the civilian setting the sand blast effect is replaced by explosive fragmentation and any surrounding environmental debris. Whilst other authors have linked energised environmental debris following blast to infection and delayed amputation, we are the first to implicate it as a causative mechanism of injury for traumatic amputation, either independently or in association with the shock front and blast wind (Khatod et al., 2003; Covey and Ficke, 2016; Rankin et al., 2020a).

The second aim of the study was to ascertain differences to the risk of injury from different loading conditions of the energised environmental debris, with reference to size and moisture content. No significant differences were seen across groups when comparing sandy gravel size (ideally distributed, small, large), moisture content (dry or saturated with water), or both. Whilst a type II error of non-significance is possible, the *P* values obtained were far from reaching significance, with values ranging from 0.194 to 1.0. As such, the data leads us to accept the null hypothesis that neither sandy gravel size nor moisture content increase the risk of traumatic amputation as occurs following high velocity sand blast in this model. Of note, the mass of sandy gravel was standardised across all experiments, irrespective of sandy gravel size. As such, it could be concluded that failure to reject the null hypothesis highlights that the total mass and dissipation of energy is the determinant factor in causing injury, as opposed to the individual size of any one piece of environmental debris.

In the present study, the 50% risk of traumatic amputation ranged from 70 m/s (95% CI 63–77 m/s) in the 0.1–0.3 mm wet sandy gravel group to 77 m/s (95% CI 69–86 m/s) in the 0.5–1.0 mm dry sandy gravel group. This compares to our previous work which showed the 50% risk of traumatic amputation in the mouse model to occur, following impact with a widely dispersed high velocity sand blast cloud, at 247 m/s (95% CI: 222–274 m/s). The same gas-gun system and standardised mass of sandy gravel was used in both experiments. In our previous work, the sandy gravel ejecta was widely dispersed to encompass a whole-body field of impact, as occurs following blast, to best recreate the boundary conditions of a blast scenario. As the present work focused on traumatic amputation in isolation, a proportionately greater mass of sandy gravel impacted with the lower limb of the specimen. As such, a greater amount of kinetic energy is expected to be imparted upon the lower limb, where kinetic energy is equal to half of an object’s mass multiplied by the velocity squared. It is therefore not unexpected that traumatic amputation was seen to occur at a lower velocity than our previous work, nor that any difference in injury risk curve distribution across groups was seen, where the sandy gravel mass across these experiments was standardised.

The current study used a focused sand blast impacting specimens from the front. This experimental setup was utilised as it most accurately allowed for traumatic amputation secondary to high velocity sand blast to occur in a reproducible manner and allowed for accurate characterisation of the injury process. It is more likely in the combat environment, however, that sand blast is encountered below the casualty and that the sand blast projectiles scatter outwards, rather than to focus on a specific target (the lower extremity as in this study). As such, the velocity values obtained from our previous work, utilising an under-body dispersing blast wave, are thought to more accurately represent the velocities required to cause traumatic amputation secondary to high velocity sand blast.

A limitation of the present study is that it was not possible to alter the standardised mass of sandy gravel, due to the experimental setup and customised sabots used in the delivery of the sand. Future work could address this limitation with further customised sabots, of differing sizes and geometry, to accommodate varying sand masses.

Whilst the current study’s findings have shown sand blast to be a mechanism of injury for traumatic amputation, scaled animal models cannot be expected to be exact replicates of what occurs in humans (Bowen et al., 1968; Bowyer, 1996; Panzer et al., 2014). Irrespective of scaling, however, this study has shown that sand blast causes significant and progressively worsening injury at high and increasing velocities, resulting in extensive soft tissue and skeletal disruption in the mouse model, and a similar effect therefore should be expected in the human. Future work reproducing high velocity sand blast could utilise human cadaveric tissue, with a focus on protective equipment which may mitigate this mechanism of injury.

This work has now allowed us to describe in detail the complete injury mechanism of traumatic amputation. Following the energy imparted by the initial shock wave (which itself may cause skeletal trauma, if the casualty is sufficiently close to the explosive), energised projectiles (sand blast; or fragmentation and other environmental debris in an urban setting) are propagated at high velocity toward the casualty. This causes initial lacerations to the skin followed by continued progression through tissue planes, as a series of microtraumas to the underlying fascia and muscular tissue occurs. With sufficient velocity the energised projectiles cause multiple fractures to the underlying skeletal structures, which compound to cause segmental and multifragmental fractures to the long bones of the limb. A critical injury point is reached, whereby the underlying integrity of both skeletal and soft tissues of the limb has been compromised. The blast wind that follows these energised projectiles completes the amputation at the level of the disruption, and traumatic amputation occurs. In cases of through-joint amputations, the energised projectiles and subsequent blast wind results in failure of the supportive soft tissues (including the ligamentous structures, but with integrity of the skeletal structures intact) to result in limb avulsion and through-joint amputation.

## Data Availability Statement

The original contributions presented in the study are included in the article/Supplementary Material, further inquiries can be directed to the corresponding author.

## Ethics Statement

Ethical review and approval was not required for the animal study because cadaveric mice were purchased as a by-product from Charles River UK. Male ex-breeder mice that had been already euthanized as per CRUK standard operating protocol, killed with a Schedule 1 procedure (CO2 asphyxiation), were subsequently used in the tests of this manuscript. These mice were accounted for under Charles River UK’s Return of Procedures Animal Use Data to the UK Home Office. As such, all animal by-product material and its use are in compliance with the UK Animal (Scientific Procedures) Act 1986.

## Author Contributions

IR, SM, and JC were involved in the conception of the study. IR and T-TN were involved in the preparation of tests, data acquisition, and in conducting the tests. IR, T-TN, and LM were involved in the data analysis. IR drafted the manuscript. All authors revised it and involved in the interpretation of the data.

## Conflict of Interest

The authors declare that the research was conducted in the absence of any commercial or financial relationships that could be construed as a potential conflict of interest.
